# A Clinical Lecture on the Value of Partial Intoxication in the Prevention of Shock during Operations

**Published:** 1881-02

**Authors:** Stephen Smith


### Selections.

### A CLINICAL LECTURE.

ON THE VALUE OF PARTIAL INTOXICATION IN THE PREVENTION OF SHOCK DURING OPERATIONS.

BY STEPHEN SMITH, A. M., M. D.

Gentlemen: This young lady is about to submit to an operation for the removal of dead bone from the region of the hip-joint, the result of long-continued disease at that articulation. I shall reserve to another occasion the discussion of some interesting questions bearing on the management of this and similar cases, and occupy the few moments allowed me in explaining what is to you the most prominent feature of her case, viz., unusual good humor and vivacity, as compared with young women brought into this room for serious operations.

As a preventive measure against shock in these cases, during an operation, partial intoxication of the patient with whiskey, brandy, or rum, will be found safe and reliable, and far preferable to quinine, opium, &c. The patient who has been laboring under great excitement in anticipation of the operation gradually becomes quite indifferent, or even bold and daring; the pulse is full and slow; the respiration undisturbed; the ether is quietly inhaled; but little, comparatively, is required; the stage of excitement is brief, or is passed without a struggle. During the operation, however prolonged, the pulse varies but slightly, unless there is a considerable loss of blood, and even in that case it maintains sufficient force to allow the operation to proceed to its completion. After the operation the pulse maintains its vigor, there is slight, if any, reaction, and the temperature remains nearly normal for the first twenty-four hours.

This practice is based upon the experience of surgeons anterior to the period of the use of anæsthetics. With them it was a well recognized fact that persons partially intoxicated at the time of the accident which necessitated the amputation, not only bore the operation with slight evidence of pain or shock, but made the best recovery. In a case of this kind I was impressed with the behavior of the patient. A man partially intoxicated entered the hospital with a crushed foot, which necessitated the amputation of the leg. He was talkative and quite indifferent both to the accident and to the proposed operation. Though the injury had occurred two hours before admission, there were but slight evidences of shock. His pulse was full and little excited, the skin was warm, the respiration undisturbed. I took advantage of his condition to amputate immediately. It was noticeable that he required but very little ether, that his pulse and respiration did not vary; in a word, that there were no evidences of shock. For twenty-four hours after the operation his pulse remained undisturbed, his skin warm and natural. He recovered within a period less than that usually occupied by similar operations.

The first case in which I purposely induced partial intoxication to prevent shock occurred many years ago. The patient was a young woman of naturally great nervous susceptibility, who was reduced to a very feeble condition by long-continued suppuration from caries of the tarsus. Amputation was advised, and although she was extremely anxious to have the operation performed, yet when the effort was made her excitement was so great that it was deemed dangerous to proceed. Twice she was placed upon the table, and the anæshetic, at one time chloroform, and ether at the other, was administered; but her pulse became so rapid and feeble, her respiration so embarrassed, her lips becoming purple, that the operation was abandoned. Finally, as a last resort, it was determined to give stimulants several hours before the operation, and until she was decidedly intoxicated. The result was most happy. When she had taken eight ounces she was in the condition of this patient, quite indifferent to the operation, her pulse was full (at 96), and her respiration tranquil. But a very small amount of ether was required, the amputation was performed, the limb was dressed, and she was placed in bed. There was no variation in the pulse or breathing during the operation, nor for twenty-four hours after. She did not discover that the amputation had been performed for sixteen hours, and, on learning the fact, was overjoyed. She made a rapid recovery.

There is another class of cases that is very favorably affected by stimulants taken during several hours preceding an operation, to the extent of partial intoxication. They are persons suffering from an enfeebled condition of the heart, and are noticeably overloaded with fat. They are very liable to succumb to even a very slight shock of the operation, when combined with the effects of the anæsthetics. The face rapidly becomes dusky, the lips purple, the respiration embarrassed, and the pulse feeble and irregular. Efforts at resuscitation sometimes prove unavailing, and the patient dies upon the table. As a preventive measure we usually give an ounce or two of whiskey just preceding the operation, and doubtless it often does prevent disaster, by arousing the heart and invigorating the circulatory organs. But such results are far more likely to be obtained if the stimulus is steadily given, in quantities suited to the condition and habits of the patient, for several hours preceding the operation. Reverting again to our patient, I must state that she is eminently a proper subject for this preparatory treatment. She entered the hospital a fortnight since, suffering from suppuration throughout the thigh, due to extensive and long-existing caries at the hip-joint. She was cadaverous in appearance, had irregular chills followed by profuse perspiration, a rapid, feeble pulse, and no appetite. Under an active tonic treatment her general condition has improved, but she is not in a state to bear safely the slightest depression from shock. The effect of the stimulant has been to give more strength and steadiness to her pulse, a warmer skin, and more cheerfulness than at any time since her admission. I do not doubt that she will bear the operation well, and that there will be no shock, unless there should be a sudden loss of a very large amount of blood. The plan which I pursue is to commence the intoxicant five or six hours before the operation, and give one, two, or three ounces every hour, according to the habits and condition of the patient. This patient required six ounces of whiskey to bring her into her present state, the first ounce having been taken six hours ago. A few days since an old drinker required sixteen ounces to induce the condition of this young woman. I have always used whiskey, and have occasionally given it in the form of milk-punch.

[It should be stated that during the operation the patient required but little ether; the pulse continued at 96, full and soft; the respiration was undisturbed. After the operation the pulse and respiration continued unaffected; there were no evidences of shock; no fever supervened; suppuration rapidly subsided; her general condition improved surprisingly, and in two weeks she resumed her hip-splint.]—*Medical Record.*

